# Can the F-Scan in-shoe pressure system be combined with the GAITRite® temporal and spatial parameter-recording walkway as a cost-effective alternative in clinical gait analysis? A validation study

**DOI:** 10.1186/s13047-023-00627-x

**Published:** 2023-05-16

**Authors:** Stephanie Speight, Sarah Reel, John Stephenson

**Affiliations:** 1 Sheffield Teaching Hospitals NHS FT, Woodhouse Clinic, 3 Skelton Lane, Sheffield, England S13 7LY; 2 University of Huddersfield, Queensgate, Huddersfield, England HD1 3DH

**Keywords:** F-Scan, GAITRite, Gait analysis, Plantar pressure

## Abstract

**Background:**

Clinical gait analysis is widely used to aid the assessment and diagnosis of symptomatic pathologies. Foot function pressure systems such as F-scan and analysis of the spatial–temporal parameters of gait using GAITRite® can provide clinicians with a more comprehensive assessment. There are systems however, such as Strideway™ that can measure these parameters simultaneously but can be expensive. F-Scan in-shoe pressure data is normally collected whilst the person is walking on a hard floor surface. The effects of the softer Gaitrite® mat upon the F-Scan in-shoe sensor pressure data is unknown. This study therefore aimed to assess the agreement between F-Scan pressure measurements taken from a standard walkway (normal hard floor), and those from a GAITRite® walkway to establish whether these two pieces of equipment (in-shoe F-Scan and GAITRite®) can be used simultaneously, as a cost-effective alternative.

**Method:**

Twenty-three participants first walked on a standard floor and then on a GAITRite® walkway wearing F-Scan pressure sensor insoles with same footwear. They repeated these walks three times on each surface. Mid gait protocols were utilised by analysing the contact pressure of the first and second metatarsophalangeal joint of the third, fifth and seventh step from each walk. For both joints, 95% Bland–Altman Limits of Agreement was used to determine a level of agreement between the two surfaces, using mean values from pressure data collected from participants who successfully completed all required walks. The intraclass correlation coefficient (ICC) and Lin’s concordance correlation coefficient were calculated as indices of reliability.

**Findings:**

ICC results for the hard surface and the GAITRrite® walkway at the first and second metatarsophalangeal joints were 0.806 and 0.991 respectively. Lin’s concordance correlation coefficient for the first and second metatarsophalangeal joints were calculated to be 0.899 and 0.956 respectively. Both sets of statistics indicate very good reproducibility. Bland–Altman plots revealed good repeatability of data at both joints.

**Conclusion:**

The level of agreement in F-Scan plantar pressures observed between walking on a normal hard floor and on a GAITRite® walkway was very high, suggesting that it is feasible to use F-Scan with GAITRite® together in a clinical setting, as an alternative to other less cost-effective standalone systems. Although it is assumed combining F-Scan with GAITRite® does not affect spatiotemporal analysis, this was not validated in this study.

## Introduction & background

Clinical gait analysis is widely used by practitioners to aid the assessment and diagnosis of symptomatic pathologies such as asymmetries, neuromuscular and musculoskeletal impairments [1]. The analysis of gait can be both subjective and objective. Subjective gait analysis uses observation alone, and as such relies on the experience, skill and knowledge of the observer [2]. Objective gait analysis uses equipment to record either force, pressure or spatio-temporal aspects of gait [1]. Whilst the measurements are recorded objectively, it is recognised that their interpretation by a clinician is subjective.

Pressure systems that record plantar pressures exist as plates, mats and in-shoe devices [3]. These systems can provide feedback to both the service user and clinician regarding the effectiveness of interventions, such as orthoses and gait re-training [3, 4]. This feedback could support the clinician’s decision-making process and improve the service user’s engagement with the management plan.

In addition to force and pressure, spatial and temporal (spatio-temporal) features of gait such as cadence, step length, stride length, base and angle of gait can provide further information regarding the pathomechanics of a person’s gait. These measurements provide the clinician with valuable diagnostic and therapeutic information that can enable gait disorders to be identified, quantified, and interventions to be accurately evaluated [5, 6].

One of the main methods in collecting spatio-temporal data is through the use of walkways. Walkways such as GAITRite® are portable, quick and simple to use and allow individuals to walk without restriction allowing a more natural gait [7]. A validation study of GAITRite® found that the data collected from individual footsteps demonstrated excellent concurrent validity, as well as a high standard of validity in cadence, speed and step length [5].

Whilst many systems exist that are capable of recording plantar pressures and spatio-temporal parameters of gait in isolation, few systems are available that can record these features concurrently. Systems such as Strideway™ incorporate both plantar pressure analysis and spatio-temporal parameters, using a tiled walkway embedded with force sensors [8]. Strideway™ provides a comprehensive evaluation of an individual’s gait pressure and spatio-temporal parameters through quantitative analysis [8]. However, there is little research available regarding the reliability or validity of Strideway™ for either research or clinical purposes [8]. Furthermore, whilst the benefits of a system capable of providing such extensive data are evident, more cost-effective alternatives may be required by practitioners.

The combination of F-Scan (in-shoe pressure measurement system) and GAITRite® (spatio-temporal measurement system) may provide an alternative to standalone high-cost systems. Numerous studies have shown the value of the F-Scan system and GAITRite® walkway in isolation, with data demonstrating reliability and validity under controlled conditions [5, 9]. However, there is a distinct lack of research investigating the effectiveness of the two systems used in combination. One concern is that the thickness of the GAITRite® walkway (5 mm) will interfere with the pressure measurements recorded by F-Scan. GAITRite® introduces a soft external surface as opposed to a hard floor typical of a clinic or gait laboratory and this variable may influence pressure recordings taken by F-Scan. Following guidelines outlined in the American Society for Testing of Materials Designation D2240 [10], a difference of 40.2 units was recorded between the two surfaces using a portable 0-100HD Shore D durometer. As there was a noticeable difference of hardness between the two surfaces, it was postulated that the GAITRite® mat may decrease pressure, change the location of pressure, or alter the timings of the pressure recorded [11]. To prevent a similar effect from the outer soles of shoes the participant wore the same footwear for walking on a hard surface and the GAITRite® walkway. Therefore, if there are any discrepancies in change in pressure this would be a result of the surface change.

This study aims to assess the agreement between F-Scan pressure measurements taken from a standard walkway (normal hard floor), and those from a GAITRite® walkway to establish whether these two pieces of equipment (in-shoe F-Scan and GAITRite®) can be used simultaneously. Specifically, it aims to determine if GAITRite® interferes with F-Scan pressure measurements when used concurrently. A measure of the reproducibility of the data will also be obtained. Gait information captured from this study may aid clinical practice in identifying a cost-effective alternative to more expensive commercially available systems.

## Method

### Participants

A convenience sample of twenty-six participants was recruited for the study from the population of a university in the UK. Ethical approval for the study was obtained from the School Research Ethics Panel at the host university.

To enter the study, participants were required to be over 18 years old, have a self-reported UK shoe size between the range of 5 and 10 and own a pair of training shoes with a secure fastening that they were prepared to wear during the study. This range of shoe sizes was chosen to capture the most commonly observed shoe sizes worn by men and women in the UK. Participants were unable to enter the study if they had a current injury, active foot disease, difficulties with balance, or depth perception.

### Data collection

Data collection took place in a gait analysis laboratory using an F-Scan Research 7.50 × and a GAITRite® walkway. The F-Scan equipment consisted of a data logger attached to the waist of the participant and two ankle receiver units linked to the in-shoe sensor insoles Prior to testing, each participant walked for five minutes whilst wearing the F-Scan equipment to allow the participant to become familiar with the device and to establish their usual walking speed. This reduced the possibility of inaccurate pressure readings being recorded from atypical gait patterns [12]. Following research recommendations [13], new F-Scan insoles were introduced after every five uses to avoid damage to the sensors housed within the in-shoe sensor insoles. In accordance with the F-Scan manual, walk calibration was performed automatically using the participants weight whilst wearing the equipment [14].

The GAITRite® walkway measured 6 m in length, 60 cm in active width (89 cm overall) and 5 mm in thickness. The walkway was not connected to the software as this was not required for the study.

**Fig. 1:**
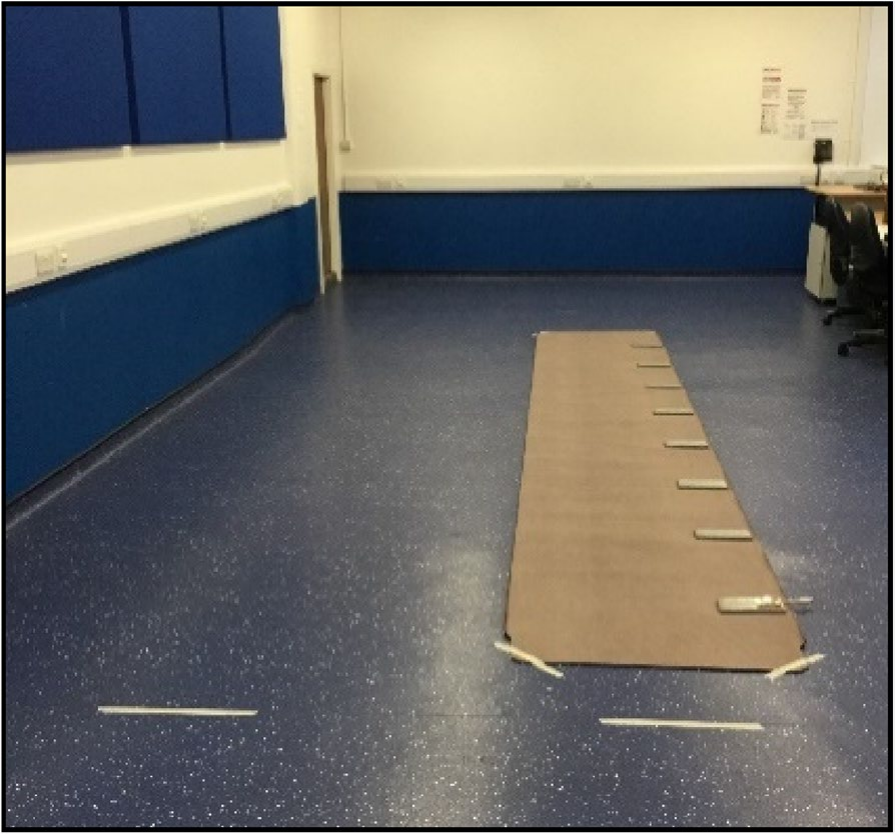
Walkway setup

Steps taken during gait initiation and gait termination are not representative of mid-gait walking steps, whereas the third and fifth steps have been found to provide an accurate representation of ‘normal’ walking patterns [15]. Therefore, a starting line was placed 30 cm in front of the GAITRite® walkway to avoid gait initiation steps and ensure the third and fifth steps were captured on the walkway. An additional start line was placed adjacent to that described above to create an adjacent walkway on the laboratory floor (Fig. 1).

The F-Scan data logger recording time was programmed at 8 s, providing sufficient time for the participants to walk the length of the walkway based upon an average cadence of 100 steps/minute [16]. This equates to approximately 13 steps being taken in the eight second recording.

The participants were asked to walk at their normal pace during the study. Participants are likely to take a different number of steps over the test distance due to varying walking speeds. Whilst research has shown that the third to fifth steps are most reflective of ‘normal’ walking in relation to pressure and force [4, 15], the selection of a single step was believed to be less representative of habitual walking than an average of steps. Therefore, an average calculation of plantar pressure recordings from the third, fifth and seventh steps was taken for each participant. For participants who had started walking with their right foot, this would equate to the second, third and fourth ground contact with the right foot. To ensure data from the same foot was collected, participants were asked to start walking with the same foot for each test condition.

Analysis was conducted at the 1^st^ and 2^nd^ metatarsophalangeal joints (MTPJs) for all participants as these are commonly investigated in foot pressure analysis particularly in diabetic care. According to research 33–35% of foot ulcerations in diabetic patients occur at the 1^st^ MTPJ joint [17].

Once the 1^st^ and 2^nd^ MTPJs on the plantar pressure recording were located, a 2 cm object box was placed in the area. This was completed manually by the researcher, a qualified podiatrist. A second podiatrist then assessed the placement of the object box to promote reliability.

To calculate the pressure at this location, the average contact pressure was calculated over the stance phase period. Contact pressure provides an understanding of the pressure acting on an anatomical structure whereas peak pressure is often used to establish the effectiveness of cushioned interventions, such as polyurethane materials [4]. Additionally, contact pressure has been found to demonstrate high retest reliability in all areas of the foot [9].

Following the five-minute ‘equipment familiarity’ period, each participant was asked to walk from the start line across the laboratory floor to the end of the room. They were then asked to walk from the start line to the end of the room on the GAITRite® walkway. This procedure was repeated three times on each surface to account for learning and fatigue effects [18, 19].

### Statistical analysis

The sample was summarised descriptively using IBM SPSS statistical software package Version 28.0.1.1. The extent and nature of any missing data was assessed. Complete case analysis was conducted following verification of low proportion of data shown to be missing completely at random. Agreement between plantar pressure recorded by the walks on the standard surface and the GAITRite® walkway was assessed using the Bland–Altman method for both joints [20], using mean values from all walks of all participants who successfully completed all required walks. The intraclass correlation coefficient (the proportion of variability between observations due to differences between walkways) and Lin’s concordance correlation coefficient [21] (a measure of the departure of the line of best fit from a 45° line through the origin) were calculated for both joints as indices of reliability.

## Results

Data were obtained from 26 subjects (6 male; 20 female) who freely gave their written informed consent to participate in this study. Each participant completed 6 recorded walks (3 per surface) giving a total of 156 walks. Inadequate calibration of the insoles led to unusable data in 4 walks. Therefore, usable data was obtained from 152 walks. Separate variance t-tests did not reveal any evidence that missing data was not missing at random and subsequent analysis was conducted on complete case analysis without imputation.

F-Scan results determined the mean plantar contact pressure recorded at the 1^st^ MTPJ by valid walks on the standard surface was 201.2 kPa (SD 68.0 kPa). The mean plantar pressure recorded by the walks on the GAITRite® walkway was 194.3 kPa (SD 78.1 kPa). The difference between the mean plantar pressure readings taken from the two surfaces was 6.9 kPa.

F-Scan results determined the mean plantar contact pressure recorded at the 2^nd^ MTPJ by valid walks on the standard surface was 241.7 kPa (SD 66.2 kPa). The mean plantar pressure recorded by the walks on the GAITRite® walkway was 241.6 kPa (SD 61.0 kPa). The difference between the mean plantar pressure readings taken from the two surfaces was 0.11 kPa.

Plantar pressure data from included walks is summarised in Table 1 below.

**Table 1:** Mean plantar pressure by surface type

*MTPJ*	*Walk number*	*Plantar pressure kPa (SD)*		
		*Standard surface*	*GAITRite® walkway*	*Both surfaces*
*1* ^*st*^	*1*	*202.1 (65.2)*	*195.5 (68.4)*	*198.8 (61.9)*
	*2*	*200.4 (66.2)*	*195.3 (84.1)*	*197.8 (64.2)*
	*3*	*201.0 (70.8)*	*192.0 (79.2)*	*196.5 (68.0)*
	*Average (all walks)*	*201.2 (68.0)*	*194.3 (78.1)*	*201.4 (65.0)*
*2* ^*nd*^	*1*	*233.5 (70.1)*	*236.7 (56.1)*	*235.2 (62.5)*
	*2*	*241.4 (67.8)*	*241.8 (63.5)*	*241.6 (64.9)*
	*3*	*250.3 (68.3)*	*246.3 (70.4)*	*248.2 (68.7)*
	*Average (all walks)*	*241.7 (67.7)*	*241.6 (62.3)*	*241.6 (64.2)*

**Fig. 2:**
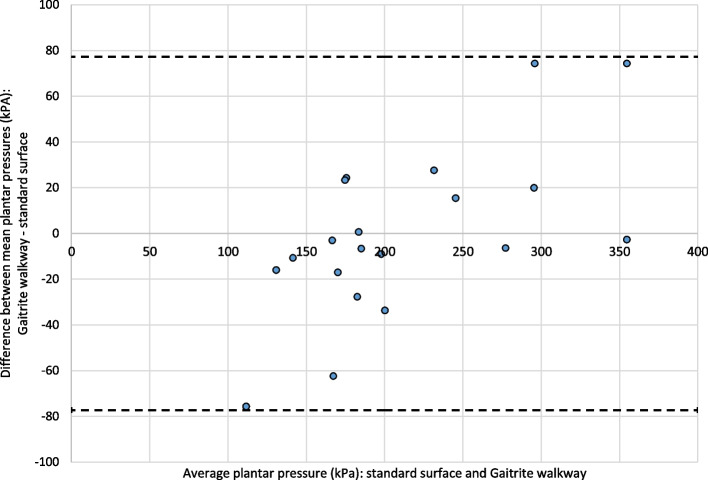
Bland–Altman plot for 1^st^ MTPJ pressures measured on the GAITRite® walkway and a standard surface

**Fig. 3:**
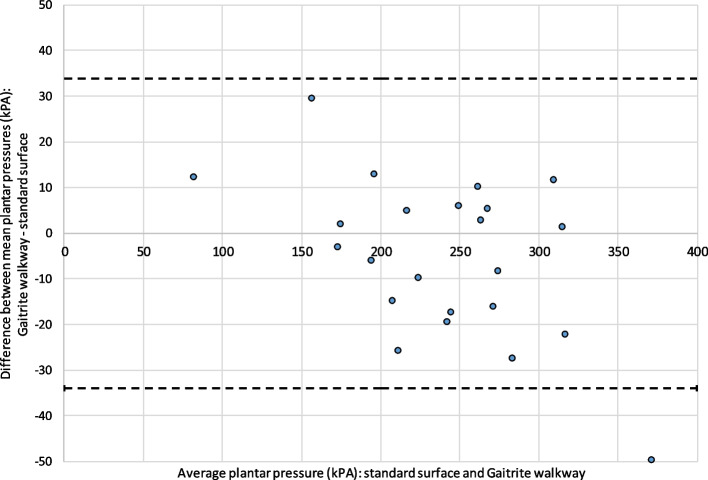
Bland–Altman plot for 2nd MTPJ pressures measured on the GAITRite® walkway and a standard surface

Bland–Altman plots of the data are illustrated in Fig. 2 (1^st^ MTPJ) and Fig. 3 (2^nd^ MTPJ). 95% limits of agreement are calculated as the mean of observed differences ± 2 standard deviations of the differences.

Values from one participant lie outside the limits of agreement on the analysis of the 2^nd^ MTPJ (no outliers were observed in the analysis of the 1^st^ MTPJ). This proportion of outlying data points is within expectations for a data set of this size. The average absolute discrepancy between values taken from the two surfaces from each participant was 19.5 kPa at the 1^st^ MTPJ and 13.9 kPa at the 2^nd^ MTPJ.

Random scatter of points may be observed, indicating no systematic difference between pairs of readings (hence a single measure of repeatability is acceptable).

No funnelling of points or other features of the data are visible; implying no evidence for a relationship between the magnitude of recorded pressure; and good levels of agreement between the two surfaces.

The intraclass correlation coefficient was calculated to be 0.806 for the 1^st^ MTPJ and 0.991 for the 2^nd^ MTPJ. Lin’s concordance correlation coefficient for the data was calculated to be 0.899 for the 1^st^ MTPJ and 0.956 for the 2^nd^ MTPJ. Both sets of statistics indicate very good reproducibility for both joints; with the 2^nd^ joint revealing particularly high levels of reproducibility.

## Discussion

F-Scan and GAITRite® are known to provide valuable gait information when used in isolation. The results of this study demonstrate negligible effects on F-Scan pressures when walking on a hard surface compared with walking on the GAITRite surface indicating that it is feasible to use the two systems in combination. Using the two systems in combination enables foot pressures and two-dimensional (spatial and temporal) gait parameters to be collected efficiently and analysed simultaneously. This provides the practitioner with correlations between gait and foot pressures and potential cause/effect relationships which may aid diagnosis and management. Using F-Scan and GAITRite® together could also provide an effective method for monitoring disease progression or the success of an intervention or management plan.

**Fig. 4:**
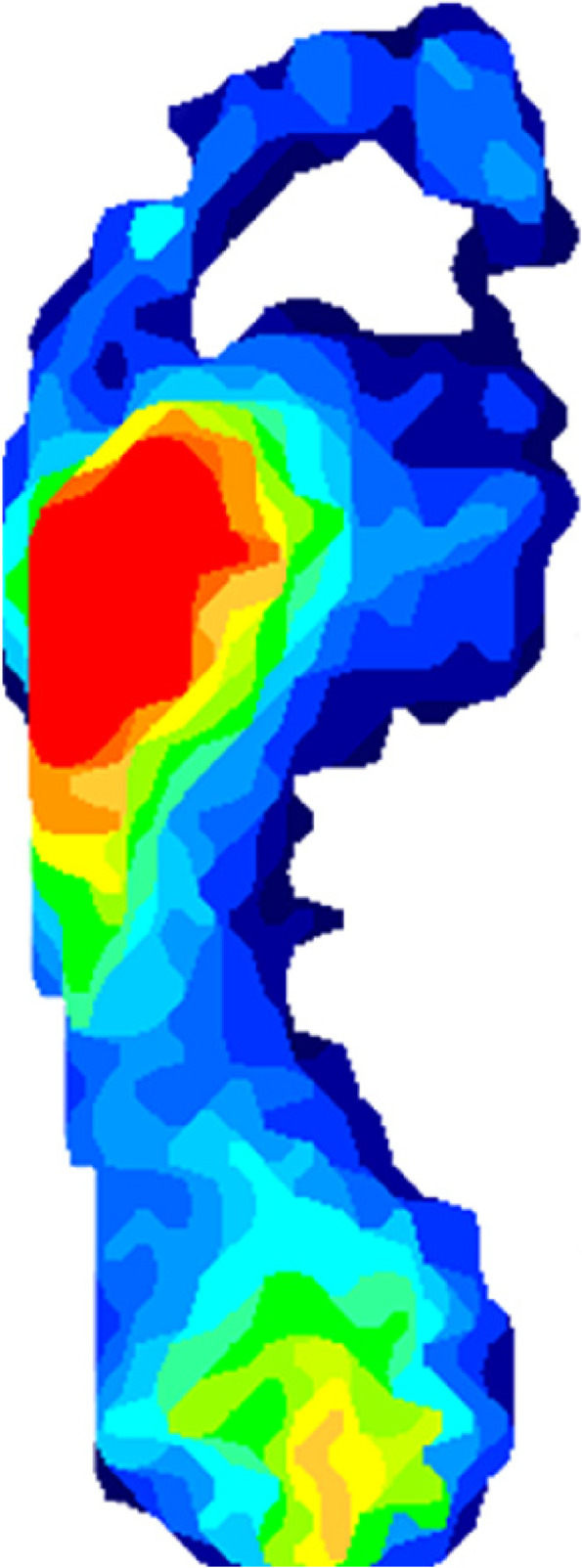
Pressure map demonstrating lateral pressure

For example, a participant in this study displayed average plantar pressure readings of 81.8 kPa (at the second MTP joint), which was lower than mean readings by a factor of 3. When investigated further, the pressure reading was a result of function, with plantar pressure for this participant being predominantly exerted through the lateral aspect of their foot (Fig. 4). There are numerous reasons for lateral foot loading when walking, one of which may relate to a reduced range of motion at the hip. Those with weak hip extensors and shortened, tight hip flexors will often show difficulty when extending the hip and consequently difficulty when toeing off from the hallux in the gait cycle [22]. If GAITRite® was used in combination with F-Scan, it may have shown a reduced step length for this participant (due to reduced hip extension) and a possible cause for the plantar pressure in this area. This provides objective, quantifiable evidence for a preferred diagnosis and enables a practitioner to treat the cause of the pathology rather than simply offloading the high-pressure area highlighted by F-Scan. Combining F-Scan and GAITRite® could provide a more comprehensive analysis in treating patients with complex pathologies.

Within-participant variation in plantar pressure recorded on the standard and GAITRite® walkways was low compared to variations recorded between participants, and variations between pressures recorded on different walks on the same walkway by the same participant. These latter sources of variation were considerable; no participant obtained constant pressure values between walks, and pressures obtained from different participants differed by a factor of 5. This finding of high inter- and intra-subject variation is consistent with supporting research [23].

Differences in mean plantar pressure between walks (for one person) on both surfaces ranged from 1 to 76 kPa at the 1^st^ MTPJ and from 2 to 39 kPa at the 2^nd^ MTPJ. Kong and De Heer [24] suggest that the ranges of differences observed at the 2^nd^ MTPJ are small, taking into consideration the natural variability of gait and the varied repeatability of plantar pressure. Research analysing the variability of plantar pressure at controlled speeds suggests that this variability can be a result of the speed at which a person walks [25]. Other studies have suggested that the natural variability of an individual’s gait could account for the frequently poor repeatability results exhibited by F-Scan [26]. However, walking speed was not controlled for in this research. The natural changes to walking speed made by a person are believed to form part of their habitual gait and consequently could contribute to associated pathology. As this research was investigating the use of these systems in clinical practice, it was deemed necessary to allow participants to walk at their naturally variable walking speed.

We expected the GAITRite® walkway would reduce the plantar pressures recorded by F-Scan. However, the extent of the reductions, and whether they would prevent the use of both systems together had not been investigated prior to this study. This difference was anticipated because the GAITRite® walkway comprises of a neoprene rubber base layer, which was expected to decelerate ground reaction forces. Price et al. [11] found that in-shoe pressure systems with foam top surfaces between 1.6 mm and 2.2 mm thick reduced plantar pressure values, when compared to the thin plastic film of F-Scan. As the GAITRite® walkway has a thickness of 5 mm, a similar or greater reduction in plantar pressure was expected. About 70% of individual pressure values recorded on F-Scan were slightly lower when the participants walked on GAITRite® compared to when they walked on the laboratory floor. However, lack of familiarity of the GAITRite® surface among the participants may have contributed to the observed differences in pressure values in earlier walks. Increased familiarity with the GAITRite® mat during the study may have reduced the differences observed between surfaces. Future research could provide participants with time to walk on the GAITRite® mat to become familiar with this surface.

## Conclusion

This study demonstrates that although plantar pressure values were expected to decrease when walking on the GAITRite® walkway rather than a hard surface, the level of agreement and consistency of measures of plantar pressures measured at both the 1^st^ and 2^nd^ MTPJs observed on the two surfaces was very high, particularly at the 2^nd^ MTPJ. These findings suggest that it is feasible to use F-Scan and GAITRite® together in a clinical setting, as an alternative to other less cost-effective standalone systems. Further research conducted on a larger and possibly more homogenous cohort of participants would be beneficial to establish whether the levels of agreement observed in the current investigation between data obtained from the two surfaces can be repeated. It is a known limitation of this study that only two areas of the foot have been investigated, therefore, further studies could involve other areas of the foot such as the heel.

## Data Availability

Additional data can be requested from the corresponding author.
